# Impact of Diabetes Status and Medication on Presentation, Treatment, and Outcome of Stage II Colon Cancer Patients

**DOI:** 10.1155/2015/189132

**Published:** 2015-05-05

**Authors:** Susie Bae, Hui-Li Wong, Jeanne Tie, Jayesh Desai, Kathryn Field, Suzanne Kosmider, Spiros Fourlanos, Ian Jones, Iain Skinner, Peter Gibbs

**Affiliations:** ^1^Royal Melbourne Hospital, Melbourne, VIC 3050, Australia; ^2^Western Hospital, Footscray, VIC 3011, Australia; ^3^BioGrid Australia, Melbourne, VIC 3050, Australia

## Abstract

Diabetes is a risk factor for colorectal cancer and several reports suggest worse cancer-specific outcomes in diabetes patients. Recent studies in multiple tumour types indicate metformin may positively impact on cancer-specific and overall survival. A population-based series of stage II colorectal cancer patients treated and followed from 2000 to 2013 were analysed for baseline characteristics, treatment, and outcomes. 1116 patients with stage II colon cancer were identified, 55.5% were male and median age was 70.9 years (range 20.5–101.2). The diabetes patients (21.6%, *n* = 241) were older than nondiabetes patients (median 74.0 versus 69.6, *p* = 0.0001). There was no impact of diabetes on cancer presentation or pathology. Diabetes patients were less likely to receive adjuvant treatment (13.7 versus 24.8%, *p* = 0.002) but were equally likely to complete treatment (69.7 versus 67.7%, *p* = 1.00). Diabetes did not significantly impact cancer recurrence (HR = 1.07, 95% CI 0.71–1.63) or overall survival (HR = 1.23, 95% CI 0.88–1.72), adjusted for age. Diabetes medication did not impact cancer recurrence or survival. Cancer presentation and outcomes in diabetes patients are comparable to those of nondiabetes patients in those with stage II colon cancer. The effect of metformin merits further evaluation in patients with colon cancer.

## 1. Introduction

A marked rise in the prevalence of diabetes has been noted in recent years [1]. Diabetes is a risk factor for colorectal cancer, with a recent meta-analysis of 24 studies reporting a relative risk of 1.26, without heterogeneity across studies [2]. However, the impact of diabetes on colorectal cancer treatment and outcomes remains uncertain. In a clinical trial enrolling predominantly patients with stage III colon cancer, diabetes was associated with an increased risk of cancer recurrence and of noncancer death [3]. A more recent cancer registry analysis found that disease-specific mortality for patients with colorectal cancer was only increased in diabetes patients with rectal cancer but confirmed an increased risk of noncancer mortality in all diabetes patients [4]. Increased overall mortality, related to an excess of deaths from cancer and noncancer causes, has been consistently reported, including a recent study by Dehal et al., who observed an increased mortality from cardiovascular disease in a group with colorectal cancer and type 2 diabetes [5].

The potential impact of diabetes medication on cancer outcomes remains an area of intense study. The multiple potential anticancer properties of metformin [6] and the mitogenicity of insulin [7] might impact cancer specific outcomes. Metformin is a biguanide, widely used in the management of type 2 diabetes. A retrospective series in patients with breast cancer found an increased rate of response to neoadjuvant therapy in those treated with metformin, prompting a large randomised study in the adjuvant setting [8, 9]. A Phase II study exploring the use of metformin in prostate cancer patients prior to prostatectomy also suggested a clinically significant impact on cancer progression [10]. A more recent study concluded that increased cumulative metformin exposure after a diagnosis of prostate cancer was associated with decreased disease specific and all-cause mortality [11]. Similar survival benefits were seen in diabetes patients with pancreatic cancer on metformin [12]. Specifically for patients with colorectal cancer several studies have reported positive effects of metformin use on overall survival in analyses combining patients with all stages of disease [13–15].

Here, we present data from a prospective database, where comprehensive data on a consecutive series of patients with colorectal cancer was entered by clinicians at four large cancer centres, allowing us to explore the impact of diabetes and diabetes medication on cancer presentation, treatment, and outcome.

## 2. Materials and Methods

Prospective data from a comprehensive colorectal cancer database from four metropolitan hospitals (Royal Melbourne Hospital, Melbourne Private Hospital, Western Hospital, and Western Private Hospital) were analysed. All patients underwent routine preoperative investigations, including computerised tomogram scans of chest, abdomen, and pelvis, with treatment and follow up based on standardised protocols. Data regarding patient status, including any recurrence or new primary cancer, was prospectively collected, with disease status documented at each follow-up visit.

Data fields examined related to initial colon cancer presentation, comorbidity including diabetes, cancer pathology, operative outcomes, adjuvant therapy use, cancer recurrence, and survival data. Baseline renal function was assessed in diabetic cohorts for underlying renal impairment. This study was approved by the Melbourne Health Human Research Ethics Committee.

We elected to focus on patients with stage II colon cancers to allow a more detailed analysis of clinicopathological characteristics and cancer-specific outcomes in a single cancer stage where there is little impact from chemotherapy. We excluded Stage I colon cancers due to the very low recurrence rate. We excluded node positive colon cancers due to the variation in delivery of adjuvant treatment (none versus fluoropyrimidine alone, versus oxaliplatin based combination treatment), and the potential confounding effect of diabetes and the frequently associated comorbidities, which might influence chemotherapy choice, chemotherapy dose, and chemotherapy completion rate. An initial search identified all patients diagnosed with stage II colon cancer between January 1, 2000, and December 31, 2013. The subset of patients with type 2 diabetes was then defined and analysed according to diabetes medication, confirmed from review of the hospital pharmacy database and individual patient files. Diabetes patients were compared with nondiabetes patients, and for diabetes patients the impact of individual antidiabetes medication was examined.

Statistical significance was analysed using SAS Enterprise Guide 6.1 (SAS institute Inc, Cary, NC, USA). Descriptive statistics including median and frequencies were used to describe the study population in each treatment category. Kaplan-Meier method was used to analyse overall survival. The Cox proportional hazards model was used for univariate and multivariate analyses of clinicopathological factors for recurrence free survival.

## 3. Results

We identified 1121 patients diagnosed with stage II colon cancer; 55.5% were males and median age was 70.9 years (range 20.5–101.2). A total of 246 patients (22.0%) with type 2 diabetes were identified. Five patients (2.0%) were excluded from analysis of the impact of diabetes medication, as at the time of colorectal cancer diagnosis their diabetes treatment could not be reliably determined from pharmacy records and chart review due to inconsistencies in data. Diabetes patients were older than nondiabetes patients (median age; 74.0 year versus 69.6 years, *p* = 0.0001).

**Figure 1:**
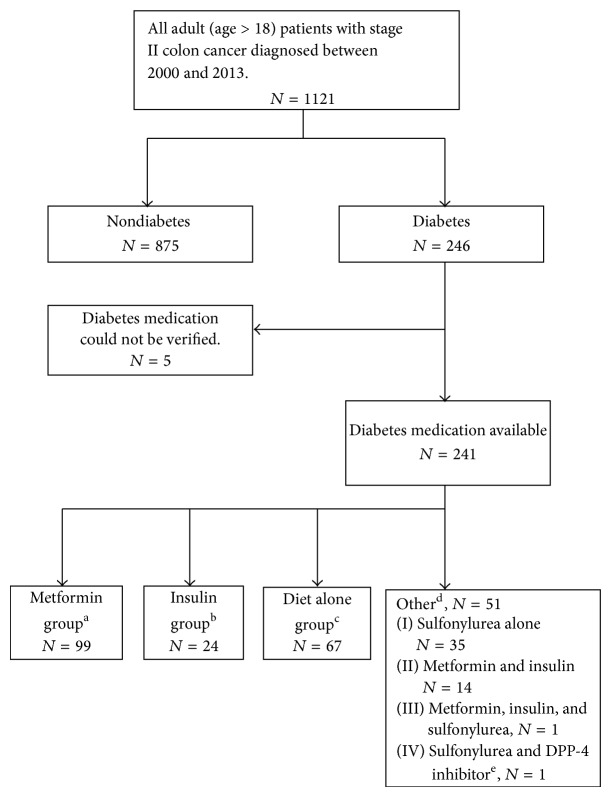
Flow chart—study population. ^a^Metformin with or without sulfonylurea; ^b^insulin with or without sulfonylurea; ^c^no diabetes medication; ^d^sulfonylurea alone or other combinations; ^e^dipeptidyl peptidase-4 inhibitor, sitagliptin used in this one patient.

Diabetes medication for the 241 patients with type 2 diabetes included in the analysis is shown in Figure 1, with 113 (46.9%) receiving metformin. Of the metformin treated patients 54 (22.4%) received metformin alone, 44 (18.3%) received metformin and a sulfonylurea and 14 (5.8%) metformin and insulin. One patient was on all three classes of drugs. Of the 128 diabetes patients not treated with metformin 67 (27.8%) were treated with diet alone, 35 (14.5%) with sulfonylurea alone, 23 (9.5%) with insulin alone and 1 (0.4%) with sulfonylurea and dipeptidyl peptidase-4 inhibitor (sitaglipin).

The impact of diabetes and diabetes medication on colon cancer pathology and presentation is shown in Tables 1 and 2. Aside from the age difference between the two groups, other clinicopathological differences were not statistically significant including the site of primary tumour. A comparison of factors that define high-risk stage II colon cancer revealed no significant association between diabetes and adverse clinicopathological features (Table 2). Microsatellite instability data were available in 580 patients (52.0%), with a comparison of diabetes and nondiabetes patients demonstrating a similar proportion of MSI-high tumours.

The operative morbidity, mortality, and adjuvant treatment details of diabetes patients, including metformin and insulin treated subsets, versus nondiabetes patients (*n* = 875) are shown in Table 3. Operative mortality, defined as death within 30 days of surgery, appears to vary according to diabetes status and within the subsets of diabetes patients; however, the small sample size and low event rate limit statistical analysis. Adjuvant chemotherapy was more commonly administered in nondiabetes patients; however, once commenced, the completion rate was similar across both groups.

Baseline renal function was available in the majority of the diabetes cohort (*n* = 234, 97.1%, Table 3). The lowest value of serum creatinine in the preceding three months prior to the diagnosis of colon cancer was taken as the baseline renal function. The mean creatinine was 87 *μ*mol/L (range 25–450) in the diabetes population. The baseline renal function was more preserved in patients treated with metformin compared to those not treated with metformin (mean creatinine; 80 *μ*mol/L (SD, 21) versus 92 *μ*mol/L (SD, 56), resp.). Using an estimated glomerular filtration rate (eGFR) of less than 60 mL/min/1.73 m^2^ as a marker for moderately impaired renal function, a greater proportion of patients in the metformin treated group had preserved renal function than the no metformin group, but the difference was not statistically significant (82% versus 75%, *p* = 0.25).

**Figure 2:**
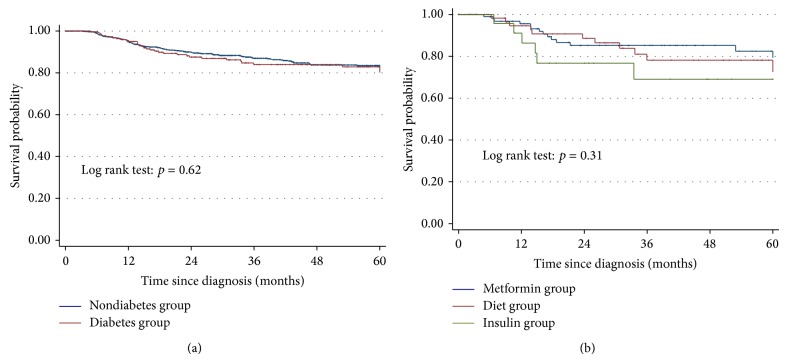
(a) Recurrence free survival of diabetes and nondiabetes stage II colon cancer patients. (b) Recurrence free survival of diabetes patients comparing diet alone group (*N* = 67) versus metformin group (*N* = 99) versus insulin group (*N* = 24).

During a median follow-up of 43.2 months, new primary colon cancers were found in 32 patients (2.9%), none of whom had diabetes (TNM stage 1; *n* = 12, TNM stage 2; *n* = 11, TNM stage 3; *n* = 4, TNM stage 4 *n* = 4; stage unknown; *n* = 1). Cox proportional-hazards regression showed no association between diabetes and the hazard of recurrence (HR = 1.07, 95% CI 0.71–1.63) or mortality (HR = 1.23, 95% CI 0.88–1.72), adjusted for age at diagnosis.  Figure 2(a) illustrates the recurrence free survival for diabetes and nondiabetes patients, with no difference seen (Log-rank test: *p* = 0.622). In univariate analysis of recurrence free survival, increased recurrence was associated with T4 stage, inadequate lymph node yield, presence of lymphovascular invasion and an emergency presentation (perforation or obstruction). These prognostic factors were retained in the multivariate regression except for the lymph node yield (Table 4).

**Figure 3:**
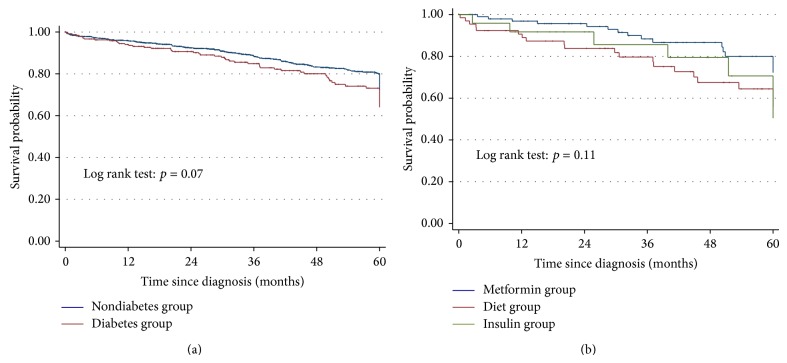
(a) Overall survival of diabetes and nondiabetes stage II colon cancer patients. (b) Overall survival of diabetes patients comparing diet alone group (*N* = 67) versus metformin group (*N* = 99) versus insulin group (*N* = 24).

For the diabetes population there was no difference in recurrence according to diabetes medication (patients receiving both metformin and insulin were excluded from the analysis of medication impact). As shown in Figure 3(a), there was a trend for a superior overall survival in nondiabetes patients compared to diabetes patients (Log-rank test: *p* = 0.066). However, this was not explained by diabetes medication.

## 4. Discussion

A better understanding of the complex relationship between diabetes and colorectal cancer development and outcome requires comprehensive and reliable data. Here we report an analysis of a large population based series that does not suggest diabetes has a major impact on colorectal cancer pathology, completion of adjuvant chemotherapy, or risk of disease recurrence for patients with stage II colon cancer. There was also no significant impact of individual diabetes medication on disease recurrence or survival in this cohort.

If diabetes or diabetes medication was to impact on the risk of colorectal cancer recurrence, this could relate to the aggressiveness of the primary cancers that develop in these patients. However, in our series we could find no association between diabetes or diabetes treatment and patient presentation with clinicopathological features that are associated with an increased risk of recurrence. This is similar to findings from previous series [13–15]. We also did not observe any impact of diabetes or diabetes medication on postoperative mortality. While mortality was numerically higher in diabetes patients and highest in insulin treated diabetes, the sample size and event rate limited our ability to show any clinically significant differences that might exist.

In our series, adjuvant chemotherapy use in diabetes patients was lower than in non-diabetes patients (13.7% versus 24.7%, *p* = 0.002), likely contributed to by the older age of diabetes patients. An excess of surgical or medical complications following the colon cancer surgery or preexisting diabetes-related illnesses, may also have contributed to less diabetes patients being fit for consideration of treatment. However, diabetes patients who received chemotherapy were equally likely to complete treatment. As the survival impact of adjuvant chemotherapy in stage II colon cancer is quite modest, the observed differences in chemotherapy administration would not be expected to show up as significant differences in rates of cancer recurrence or overall survival.

We did not observe any impact of diabetes on colon cancer recurrence. There was also no observed impact of diabetes medication on risk of cancer recurrence. However, other well-known prognostic factors for cancer recurrence such as T4 stage, inadequate lymph node yield, presence of lymphovascular invasion, and emergency presentation were found to be of significance. There is a modest trend for better outcomes for metformin treated patients and worse outcomes for insulin treated patients, compared to patients on diet control alone. While there was a trend for diabetes patients to have an inferior overall survival (Log rank test; *p* = 0.052), this was partly due to the elevated age in this population, as seen from the Cox proportional-hazards regression adjusting for age.

There was no evidence for diabetes medication impacting overall survival. This finding is in contrast with three previous single-institution studies where metformin use was found to be associated with better overall survival [13–15]. However, each of these studies included colon and rectal cancers of all stages, assessing the survival outcomes by adjusting for pathologic variables including stage at diagnosis, rather than undertaking a stage-by-stage comparison. Potential explanations for the discordance between these studies and our series include a variable effect dependent on site of disease (colon versus rectum) or on disease stage at diagnosis. Notably, in the study by the Dutch group, the impact of diabetes on outcomes was limited to patients with rectal cancer [4]. Certainly if the difference we observed in chemotherapy administration rates for stage II colon cancer was reproduced in the stage III cases (where chemotherapy impact is far greater) in these series then worse cancer specific survival could be expected in the subset of diabetes patients due to less of these receiving adjuvant therapy.

In an analysis of a stage III colon cancer clinical trial cohort, an increased risk of cancer recurrence was found in diabetes patients [3]. Unfortunately data related to patient height and weight were not available, which would have been of relevance given the greater number of diabetes patients that would be expected to be overweight, which can impact chemotherapy dosing, and may independently be associated with worse outcomes [16]. Also, in this unplanned post hoc analysis data related to diabetes status were extracted based partly on recorded medication use, which would be expected to miss many cases of diabetes.

When the impact of diabetes medication on overall survival was analysed in our series, a trend was observed in favour of metformin treated patients. Such analysis is confounded by patient factors associated with metformin administration that also are associated with survival. While we found no significant difference in renal function, we did observe that metformin treated patients were younger and more likely female. Reduced survival in insulin treated patients would not be unexpected due to insulin use being associated with obesity and poor diabetes control, both of which would predict increased noncancer deaths. Only one patient was noted to be on a newer diabetes medication, DPP-4 inhibitor, which has only recently become widely available in Australia.

There are several strengths to our data including the prospective nature of comprehensive data collection from the time of diagnosis through to recurrence and death, the standard approach to patient workup and treatment, and the relatively large sample size. Diabetes status was collected at diagnosis, along with diabetes medication, and detailed data on cancer recurrence and new primary cancers was available. We elected to examine stage II colon cancer for several reasons. We felt that examining one stage of disease rather than a combined analysis of all stages would allow a detailed analysis of cancer pathology, and also make interpretation of outcomes more reliable. Also, if diabetes proved to be an adverse prognostic factor then this could be added to the current list of high-risk features that guide adjuvant chemotherapy decision-making for stage II cases. Finally, given the low rate of usage and the marginal impact of adjuvant therapy on stage II colon cancer outcomes, there is minimal confounding due to any differences in anticancer treatment received, dosing, or treatment duration.

The weaknesses of our study include that the relatively low rate of recurrence in stage II colon cancer makes it more difficult to detect small but clinically significant differences between groups. Also despite a relatively large overall sample size the number of metformin and insulin treated patients was quite modest, particularly once patients on multiple medications were excluded.

## 5. Conclusions

Our study demonstrates that, for patients with stage II colon cancer, diabetes is not associated with clinicopathological factors that are known to bestow an increased risk of cancer recurrence. We found a difference in the rate of administration of adjuvant chemotherapy, which has implications for series examining outcomes in node positive cancers given adjuvant therapy has far greater impact on survival outcomes in these patients. We did not observe any significant associations between diabetes or diabetes medication and risk of cancer recurrence or death. Future prospective, randomised studies will further clarify the impact of diabetes medication on colon cancer outcomes. The results of such studies are eagerly awaited, both for colorectal cancer and for other cancers where metformin in particular shows promise as an anticancer agent.

## Figures and Tables

**Table 1:** Clinicopathological features of nondiabetes versus diabetes patients.

	All patients *N* = 1116	Patients without type 2 diabetes *N* = 875	Patients with type 2 diabetes *N* = 241
Median age in years (range)	70.9 (20.5, 101.2)	69.6 (20.5–101.2)	74.0 (26.5–93.3)
Sex			
Female (%)	497 (44.5%)	400 (45.7%)	97 (40.3%)
Male (%)	619 (55.5%)	475 (54.3%)	144 (59.7%)
Site			
Right colon (%)	533 (47.8%)	407 (46.5%)	126 (52.3%)
Left colon (%)	583 (52.2%)	467 (53.4%)	115 (47.7%)
T4 staging (%)	176 (15.8%)	131 (15.0%)	45 (18.7%)
<12 lymph nodes examined	275 (24.6%)	208 (23.8%)	67 (27.8%)
Poor differentiation (%)	225 (20.2%)	170 (19.4%)	55 (22.8%)
Lymphovascular invasion	266 (23.8%)	211 (24.1%)	55 (22.8%)
MSI status (%)			
MSI-stable	441 (39.5%)	351 (40.1%)	90 (37.3%)
MSI-high	114 (10.2%)	92 (10.5%)	22 (9.1%)
Not done	536 (48.0%)	413 (47.2%)	123 (51.0%)
Unknown	25 (2.2%)	19 (2.2%)	6 (2.5%)
Emergency presentation^a^	158 (14.2%)	122 (13.9%)	36 (14.9%)

^a^Emergency presentation includes bowel perforation or bowel obstruction.

**Table 2:** Pathologic features of diabetes patients: metformin and insulin treated subsets.

	Metformin group *N* = 99	Insulin group *N* = 24	*p * value
Median age in years (range)	73.4 (51.1–93.3)	71.8 (39.6–85.3)	0.28
Sex			
Female (%)	41 (41.4%)	3 (12.5%)	0.008
Male (%)	58 (58.6%)	21 (87.5%)	
Site			
Right colon (%)	59 (60%)	14 (58%)	1.0
Left colon (%)	40 (40%)	10 (42%)	
T4 staging (%)	17 (17.2%)	4 (16.7%)	1.0
<12 lymph node sampling	28 (28.3%)	7 (29.2%)	0.09
Poor differentiation (%)	25 (25.2%)	2 (8.3%)	0.7
Lymphovascular invasion	21 (21.2%)	4 (16.7%)	1.0
MSI status (%)			
MSI-stable	36 (36.4%)	9 (37.5%)	1.0
MSI-high	18 (18.2%)	4 (16.7%)	
Not done	43 (43.4%)	11 (45.8%)	
Unknown	2 (2.0%)	0	
Emergency presentation^a^	11 (11.1%)	6 (25.0%)	0.09

^a^Emergency presentation includes bowel perforation or bowel obstruction.

**Table 3:** Operative morbidity and mortality, adjuvant treatment commenced, and completed details of nondiabetes versus diabetes patients, including metformin and insulin treated subsets.

	No diabetes *N* = 875	Diabetes *N* = 241	Metformin group *N* = 99	Insulin group *N* = 24
Renal function				
*μ*mol/L, mean Cr^a^	N/A	87	80	92
*n* (%) eGFR < 60^b^	N/A	49 (22%)	17 (18%)	32 (25%)
Surgical complications, *n* (%)	185 (21.1)	67 (27.8)	31 (31.3)	7 (29.2)
Operative mortality, *n* (%)	14 (1.6)	5 (2.1)	1 (1.0)	1 (4.2)
Adjuvant chemotherapy commenced, *n* (%)	217 (24.8)	33 (13.7)	11 (11.1)	4 (16.7)
Adjuvant chemotherapy completed, *n* (%)	593 (67.8)	168 (69.7)	63 (63.4)	18 (75)

^a^Serum creatinine.

^b^Number and percentage of patients with estimated glomerular filtration rate less than 60 mL/min/1.73 m^2^.

**Table 4:** Univariate and multivariate analyses for recurrence-free survival.

Clinicopathological variable^a^	Univariate model			Multivariate model		
	HR^b^	95% CI^c^	*p*	HR^b^	95% CI^c^	*p*
Age						
≤60 years	1.00	0.87–2.02	0.184	1.00	0.86–2.14	0.193
>60 years	1.33			1.35		
Diabetes						
No diabetes	1.00	0.74–1.60	0.667	1.00	0.65–1.49	0.93
Diabetes	1.09			0.98		
T stage						
T3	1.00	1.65–3.41	<0.001	1.00	1.36–2.94	<0.001
T4	2.37			2.00		
Lymph node yield						
≥12	1.00	1.01–2.02	0.043	1.00	0.89–1.89	0.169
<12	1.43			1.30		
Lymphovascular invasion						
Absent	1.00	1.57–3.12	<0.001	1.00	1.43–2.89	<0.001
Present	2.21			2.03		
Emergency presentation						
No	1.00	1.21–2.75	0.004	1.00	1.07–2.49	0.022
Yes	1.82			1.63		

^a^Microsatellite instability status was not included in the regression models for lack of complete data (complete data in 52% of patients).

^b^Hazard ratio.

^c^Confidence interval.
